# Absorptive Power of the Skin

**Published:** 1863-10

**Authors:** 


Absorptive power of the Skin.—Dr. Parisot has addressed two papers to the Academy of Sciences, on the absorptive power of the skin, for the purpose of testing the efficacy of baths. The following substances, iodide of potassium, the yellow cyanide and chlorate of the same, sulphate of iron, belladonna, foxglove and rhubarb, he has tried upon himself, and some of them upon patients whose state required it. The baths were taken early in the morning, fasting, the temperature of the water ranging between 28 and 30 deg. Centigrade (80–86 deg. F.), that of the air being between 18 and 27 (62–79 F.) The duration of the immersion was from one to two hours for Dr. Parisot himself, and from thirty minutes to one hour for his patients. The results obtained were, that salts and the coloring matter of rhubarb dissolved in water are not absorbed by the skin, and that vegetable poisons, such as foxglove and atropine, dissolved in water, are also not absorbed by the skin, because after a lengthened stay in the baths no symptoms of poisoning are experienced. Dr. Parisot's experiments on water, chloroform and ether are peculiarly striking. The body of an infant which had died twelve days after birth, was laid in a bathing tub, the water of which was incessantly renewed by a permanent influx and efflux of the liquid—the head was kept above water. At the end of twenty four hours, the body, which weighed 2,050 grammes (4 pounds and 1¾ ounces) before the immersion, weighed 2,055 grammes. Being afterwards left for twenty-four hours in the laboratory, and then weighed, it was found to have lost the five grammes it had gained in the bath. Now the epidermis has a sebacious coating everywhere except on the palms of the hands and the soles of the feet—it remained therefore to be seen whether the imbibition had been effected throughout, or only at the hands and feet. In another series of experiments the palms and soles of certain subjects were coated with turpentine, while those of others were not—and the result was, that the former did not increase in weight by immersion, while the latter did. Hence the palms of the hands and the soles of the feet must be considered the only points of the human body through which imbibition can take place, they being deprived of that sebaceous covering which acts as a protecting varnish all over the rest of the body. Chloroform, alcohol and ether dissolve the sebaceous matter more or less completely, and hence the substances dissolved in them may penetrate to the derma. Dr. Parisot dissolved five centigrammes (one grain) of atropine in twenty grammes (five drachms) of chloroform; he then steeped cotton in the solution and applied it to the forehead of a person; the extension of the pupil became visible after the lapse of three minutes—two minutes later it was complete and equal on both sides—after a quarter of an hour the skin was found to be red and burning. All these symptoms disappeared in the course of an hour. Substituting alcohol for chloroform, the action was greatly retarded, the dilatation not taking place until half an hour's application, and the redness and heat of the skin were hardly perceptible. Atropine dissolved in water slightly acidulated with acetic acid produced no effect whatever.—*Med. & Surg. Reporter.*

